# Cases of Monkeypox show highly-overlapping co-infection with HIV and syphilis

**DOI:** 10.3389/fpubh.2023.1276821

**Published:** 2024-01-05

**Authors:** Lin Jia, Benyong Yan, Yuan Fang, Xiaodong Yang, Han Jia, Mei Zhang, Shuang Li, Yang Zhang, Wen Wang, Caiping Guo, Tong Zhang, Xiaojie Huang, Taiyi Jiang

**Affiliations:** Clinical and Research Center for Infectious Diseases, Beijing Youan Hospital, Capital Medical University, Beijing, China

**Keywords:** Monkeypox, transmission routes, clinical features, sexually transmitted infection, viral loads

## Abstract

**Purpose:**

Ongoing Monkeypox (MPX) outbreaks in countries outside Africa have unique characteristics. However, data on cohorts of confirmed cases in China is limited. The study provides important epidemiological, diagnostic, and clinical information about this disease in China.

**Methods:**

We report a series of Chinese individuals with confirmed MPX infections identified at Beijing Youan Hospital (China) from June 10 to July 15, 2023. Samples were taken from the skin, anus, throat, and blood. An epidemiological questionnaire was used to collect demographic and clinical data. Further, we compared the MPX viral (MPXV) loads across different anatomical sites.

**Results:**

66 samples were collected from 20 patients, all of whom were cisgender men. Median patient age was 29 years. Notably, 19 (95%) patients reported unprotected sexual encounters with men in the preceding month, and 13 (65%) were human immunodeficiency virus (HIV)-positive. Among those with HIV, 12 (92%) were receiving antiretroviral therapy, and 11 (85%) had well-controlled infections (HIV viral load <40/mL). The median CD4+ T cell count was 667 cells/mm^3^. In the HIV-negative group, three (43%) patients were taking preexposure prophylaxis. Fifteen patients (75%) had concurrent sexually transmitted infections (50% had syphilis and 65% had HIV) and eight (40%) had HIV and syphilis co-infection. MPXV loads were significantly higher in samples from the skin (cycle threshold value [Ct value]: 19·0) and anus (Ct value: 23.0) compared to samples from the throat (Ct value: 31.0) or blood (Ct value: 34.5). All patients had skin lesions (85% of whom presented with anogenital lesions). Common systemic symptoms included fever (85%) and lymphadenopathy (55%). The median incubation period was 8 d [interquartile range (IQR): 6–16 d]. The median time from the onset of skin lesions to scab removal was 14 d (IQR: 10–16 d). No deaths or severe cases were reported.

**Conclusion:**

MPXV primarily affects young homosexual men. The high MPXV viral loads in skin and anal lesions indicate that transmission most likely occurs through direct and close body contact. This study also reports high rates of HIV and syphilis co-infection. Therefore, preventive efforts should focus on homosexual men.

## 1 Introduction

Monkeypox (MPX) is an infectious disease caused by the Monkeypox virus (MPXV), an enveloped double-stranded DNA virus of the Orthopoxvirus genus in the Poxviridae family. There are two different clades exist: clade I and clade II. It can cause a painful rash, enlarged lymph nodes and fever. Most people fully recover, but some get very sick. The first reported human case of MPX was a nine-month-old boy in the Democratic Republic of the Congo in 1970. MPX can spread from person to person or occasionally from animals to people. It can be transmitted to humans through physical contact with someone who is infectious, with contaminated materials, or with infected animals ^(1)^. Following eradication of smallpox in 1980 and the end of smallpox vaccination worldwide, it steadily emerged in central, east and west Africa. Since May 2022, the emergence of MPXV outside Africa has rapidly led to global outbreaks of MPXV infections in humans. On June 23, 2022, the World Health Organization declared MPXV an “evolving threat of moderate public health concern,” which served as a global wakeup call. As of July 25, 2023, there have been 88,600 confirmed cases reported from 113 countries (1). By July 14, 2023, the Chinese Center for Disease Control and Prevention had reported over 106 cases across six provinces (45 cases in Beijing) (2). The current spread of MPX appears to predominantly occur through close or sexual contact, mainly among men who have sex with men (MSM), indicating likely human-to-human transmission. Prior studies have shown that 28–47% of confirmed cases are also living with human immunodeficiency virus (HIV) (3). Cohort data from the United States, Germany, and Spain show that approximately 50% of MPXV infections are co-infected with at least one other sexually transmitted infection (STI), including syphilis, herpes, gonorrhea, chlamydia, and *Mycoplasma genitalium* (4). Here, we present the epidemiological, diagnostic, and clinical characteristics of 20 patients with confirmed MPX infections at a referral hospital in China. Our study presents characteristics of MPXV infections that have not been described in China since 2022.

## 2 Materials and methods

### 2.1 Study design

We conducted an observational study of MPXV cases (confirmed using a positive viral polymerase chain reaction [PCR] test for MPX from any anatomical site) diagnosed between June 10, 2023, and July 15, 2023, at Beijing Youan Hospital, a referral center for MPXV infection in Beijing, China.

### 2.2 Procedures

All patients underwent standard medical examinations. During their initial visit, samples for the MPXV-specific PCR assay, including blood, skin lesion, throat, and anal swabs, were collected and tested at the Beijing Centers for Disease Prevention and Control. MPX cases were confirmed by positive PCR test results [cycle threshold value (Ct) <40] for any type of specimen. The treating physician collected information on the demographic, clinical, and behavioral characteristics of each patient. In addition, all patients were tested for HIV and syphilis infections. In accordance with the recommendations of the Chinese Center for Disease Control and Prevention for controlling MPXV outbreaks (2), health workers used facemasks and gloves to provide patient care in the isolation unit, including reducing pain and preventing dehydration. Desquamation of all lesions allowed the patient to be discharged. Complications of illness and patient prognoses were also recorded.

### 2.3 Variables

A structured epidemiological questionnaire was administered, which included sex, age, vaccination against smallpox, sexual orientation, HIV status, preexposure HIV prophylaxis therapy, date of symptom onset, signs, and symptoms at presentation, sexual activity, and number of sexual partners during the 30 d prior to symptom onset.

We recorded clinical data (including symptoms and complications of illness), as well as MPXV loads, which were given as Ct values from the skin, anal, throat, and blood samples. Further, instances of concomitant STIs with MPX were recorded.

### 2.4 Ethical considerations

Patients provided consent for the use of their medical data and the publication of anonymized clinical details and images. The study protocol was approved by the Ethics Committee of Beijing Youan Hospital (China) (approval number: LL-2023-035-K), and voluntary informed consent was obtained from each participant.

### 2.5 Statistical analysis

Qualitative variables were documented as absolute numbers and percentages. Quantitative variables are presented as means ± standard error or median with interquartile range (IQR). The proportions of MPXV-PCR-positive and MPXV-PCR-negative samples among different anatomical sites were compared using Chi-square tests. The Ct values of the MPXV viral loads among the anatomical sites were compared using the Kruskal-Wallis test. Statistical analyses were performed using IBM SPSS Statistics version 28.0, and the significance threshold was set at *p* < 0.05.

## 3 Results

Between June 10, 2023, and July 15, 2023, Beijing Youan Hospital’s HIV clinics attended to 7,985 patients with HIV infection, and the HIV Inpatient Department cared for 114 patients. Twenty patients were confirmed to have an MPX infection, with at least one positive PCR result (Table 1). All patients were cisgender MSM, with a median age of 29 years (IQR: 26–32 years). None of the patients had been vaccinated against smallpox. Furthermore, 65% of patients (*n* = 13) were individuals with HIV, of whom 92% (*n* = 12) were on antiretroviral therapy. Eleven (85%) patients showed HIV suppression with undetectable viremia (<40 copies/mL), and the median CD4+ lymphocyte count was 667 cells/mm^3^ (IQR: 404–902). This period also saw the diagnosis of a new HIV case. Among individuals without HIV, 43% (three cases) were on daily preexposure prophylaxis against HIV infection with tenofovir disoproxil fumarate/emtricitabine. Furthermore, 19 (95%) patients reported having unprotected sexual encounters with men in the past month, with some patients engaging in high-risk behaviors with multiple partners.

**Table 1:** Patients’ characteristics.

	Participants (*n* = 20)
Sex	
Male	20 (100%)
Age, years (Median [IQR])	29 (26–32)
Range	24–39
Anti-smallpox vaccination	0
Suspected transmission route	
Sex with men	19 (95%)
Kiss with men	1 (5%)
Number of sex partners in the past month	
Median (IQR)	1 (1–2.5)
Attendance at a sex-on-site event in the past month	
Yes	5 (25%)
No	15 (75%)
HIV co-infection	
Yes	13 (65%)
No	7 (35%)
Syphilis co-infection	
Yes	10 (50%)
No	10 (50%)
Preexposure prophylaxis in HIV-negative patients	
Yes	3 (15%)
No	4 (20%)
Medical setting at first admission	
Infectious diseases department	1 (5%)
Fever clinics	3 (15%)
Sexual health clinic	8 (40%)
Dermatological clinic	8 (40%)

IQR, interquartile range; HIV, human immunodeficiency virus.

**Figure 1:**
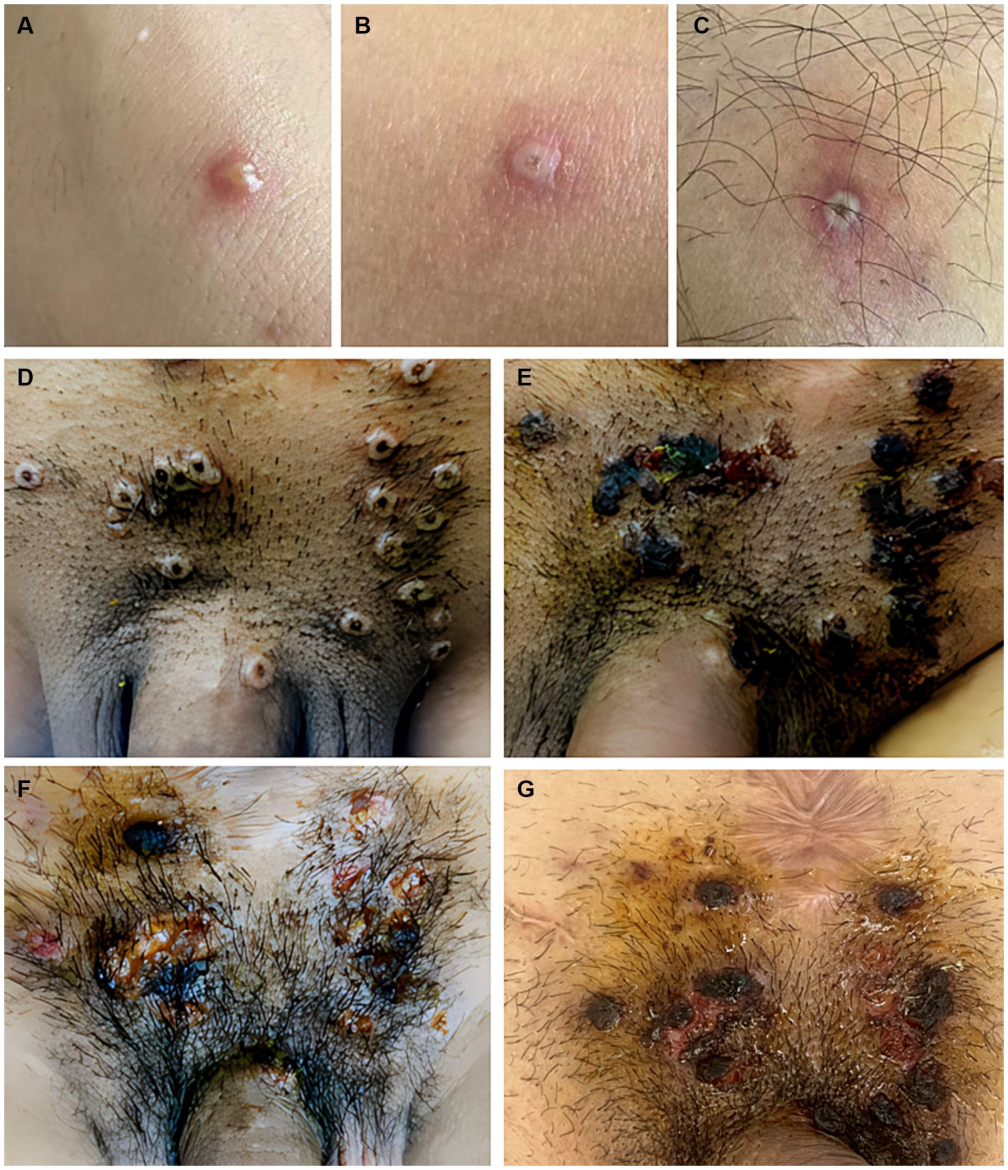
Skin features include **(A)** pustular or vesicular lesions in the forearm, **(B,C)** sub-centimeter vesicles and papulovesicles with central necrosis involving the legs, and **(D–G)** lesions in the perineum. The vesicles develop a central umbilication that is initially subtle and gradually enlarges as the color darkens to brown. Eventually, the lesions ulcerate, leaving a central necrotic crust that entirely covers the lesion. After the crust becomes detached, the underlying skin presented with residual erythema or variable post-inflammatory hyperpigmentation or hypopigmentation.

**Figure 2:**
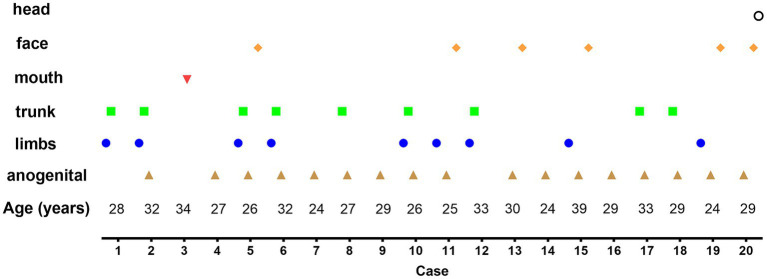
Distribution of lesions in each confirmed Monkeypox (MPX) case. Anogenital (85%), trunk and limbs (60%), face and mouth (35%), and head (5%).

Patients presented with various skin lesions (Figure 1). Notably, 85% of patients had anogenital lesions, 60% had trunk and limb lesions, 35% had face and mouth lesions, and 5% had head lesions (Figure 2).

**Figure 3:**
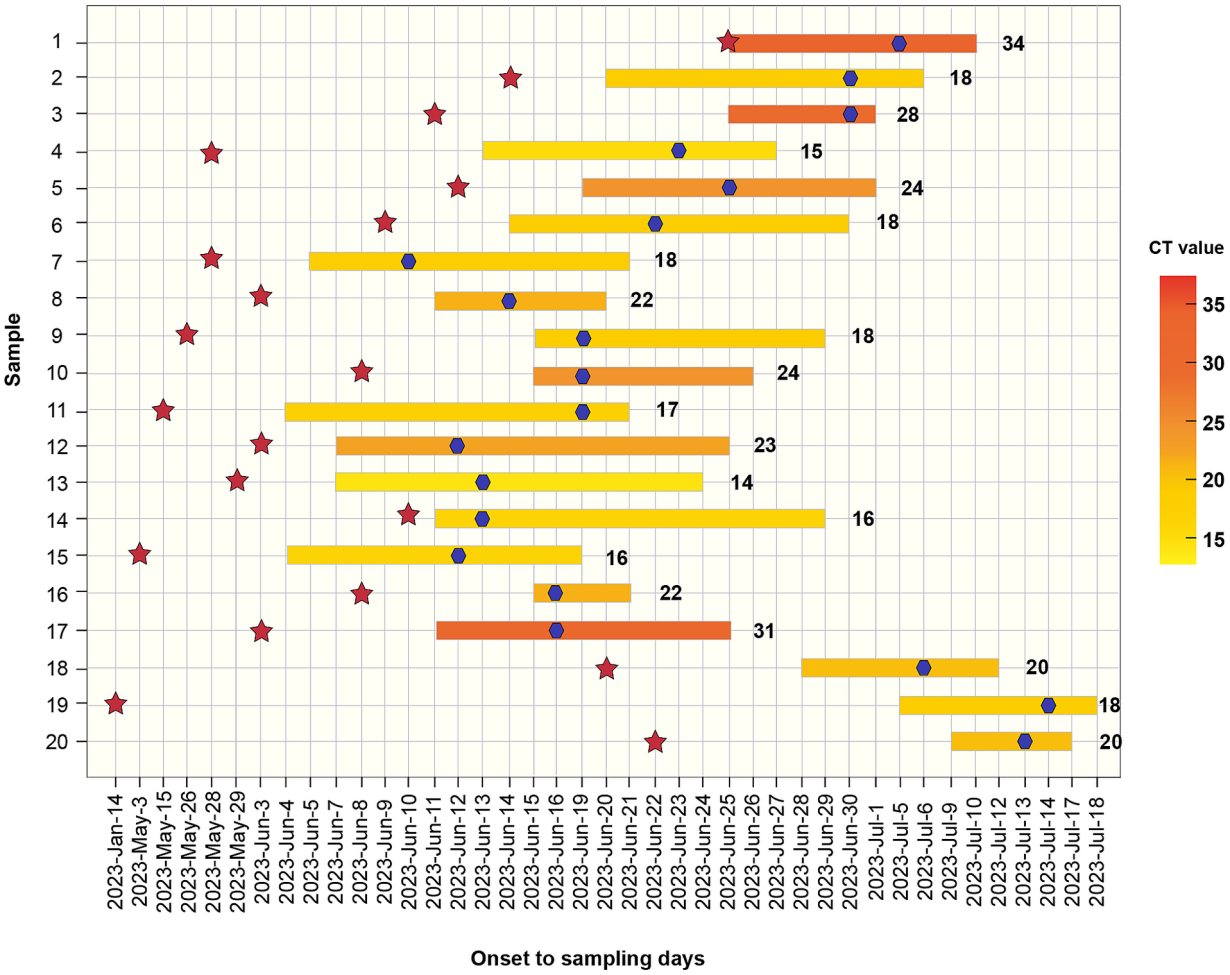
Distribution of Monkeypox cases by symptom onset from June to July 2023. Bars represent the onset date and the desquamation date of all lesions. The cycle threshold value (Ct value) is indicated in a yellow scale for every case and their identification is also shown. Red stars represent the participation in sexual contact events. Blue stars indicate the date of sampling.

Overall, 65% of patients initially presented with fever (*n* = 13), while 35% reported skin lesions as their first symptom (*n* = 7). None of the patients had traveled to areas with known infections or had direct contact with infected foreign individuals before the onset of symptoms. Common systemic symptoms included fever (85%) and lymphadenopathy (55%). Other clinical presentations included tonsillitis (5%), diarrhea (5%), myalgia (5%), anal pain (25%), oral ulcers (5%), urodynia (5%), and penitis (25%) (Table 2). The median time between exposure and symptom onset was 8 d (IQR, 6–16 d). The median time from initial symptom onset to hospital admission was 6 d (IQR: 4–9 d). The median time from the skin lesion onset to desquamation of skin lesions was 14 d (IQR, 10–16 d) (Figure 3).

**Table 2:** Clinical presentation of patients.

	Participants (*n* = 20)
Clinical features	
Skin lesions	20 (100%)
Fever	17 (85%)
Lymphadenopathy	11 (55%)
Tonsillitis	1 (5%)
Diarrhea	1 (5%)
Myalgia	1 (5%)
Anal pain	5 (25%)
Oral ulcer	1 (5%)
Urodynia	1 (5%)
Penitis	5 (25%)
Prodromes (general signs before skin lesions)	
Yes	13 (65%)
No	7 (35%)
Skin lesions number at time of MPX diagnosis	
1	0
2–5	3 (15%)
6–10	12 (60%)
11–50	5 (25%)
>50	0
Skin lesions localization at time of MPX diagnosis	
Body	9 (45%)
Face	8 (40%)
Palms or soles	9 (45%)
Genitals	13 (55%)
Anal margin	4 (10%)

MPX, Monkeypox.

**Figure 4:**
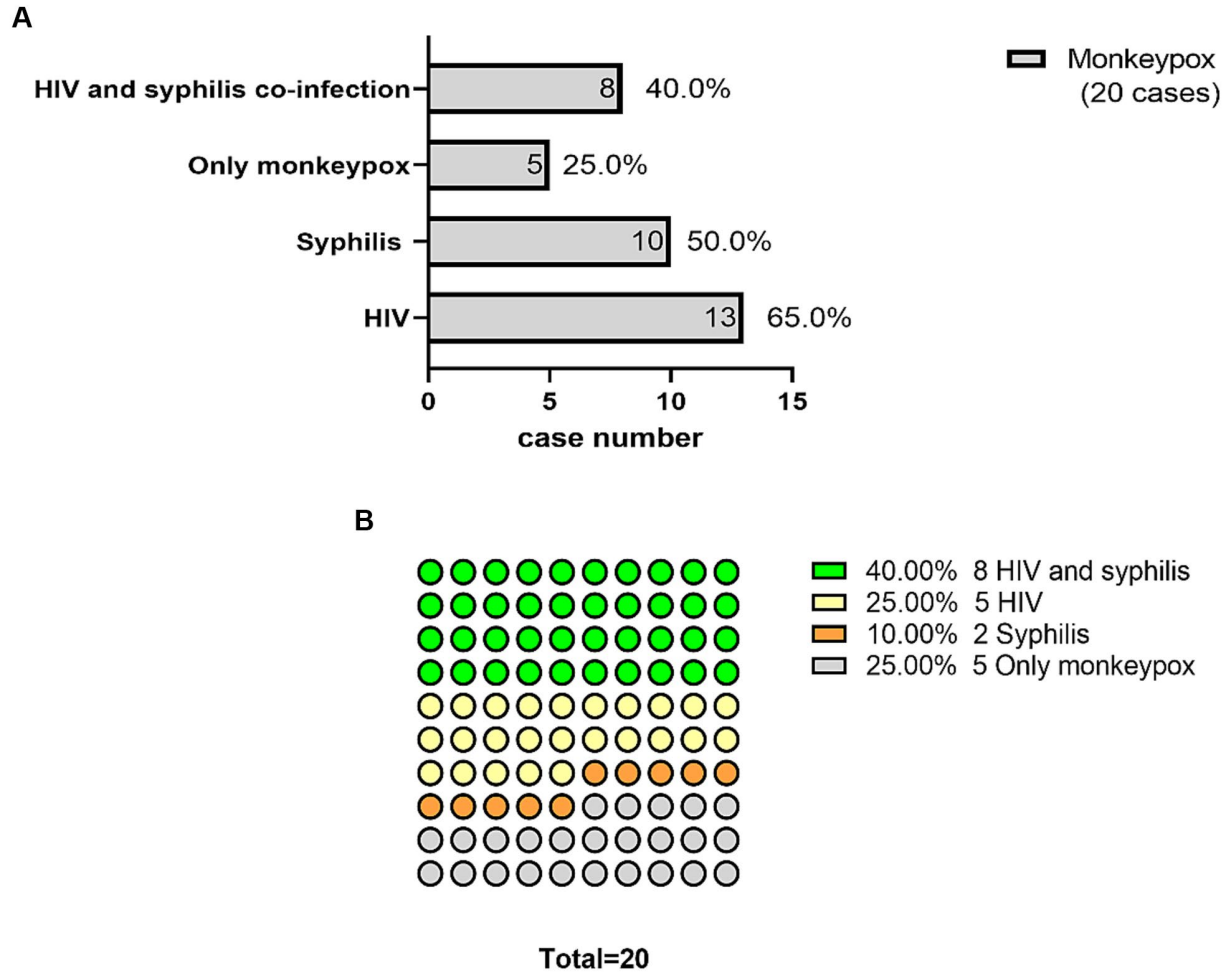
Monkeypox (MPX) overlapped with HIV and syphilis co-infection. Fifteen patients (75%) were diagnosed with concomitant sexually transmitted infections (50% had syphilis and 65% had human immunodeficiency virus [HIV]). Eight (40%) had co-infections of HIV and syphilis **(A)**. MPX co-infected with both HIV and syphilis (40%) occurred at a higher rate than MPX infection alone (25%), followed by MPX co-infected with HIV (25%) and MPX co-infected with syphilis (10%) **(B)**.

Among the 20 patients with confirmed MPX, 15 were diagnosed with syphilis or HIV. Comparing the frequencies between the two groups one with only MPX (25%) and the other co-infected with syphilis or HIV (75%) there was a significant difference (*p* = 0.002), as determined by the chi-square test. Fifteen patients (75%) were diagnosed with concomitant STIs (50% had syphilis and 65% had HIV), and eight (40%) patients had HIV and syphilis co-infection (Figure 4A). The incidence of MPX co-infection with both HIV and syphilis (40%) was higher than that of MPX infection alone (25%). This was followed by MPX co-infection with HIV (25%) and MPX co-infection with syphilis (10%) (Figure 4B).

**Figure 5:**
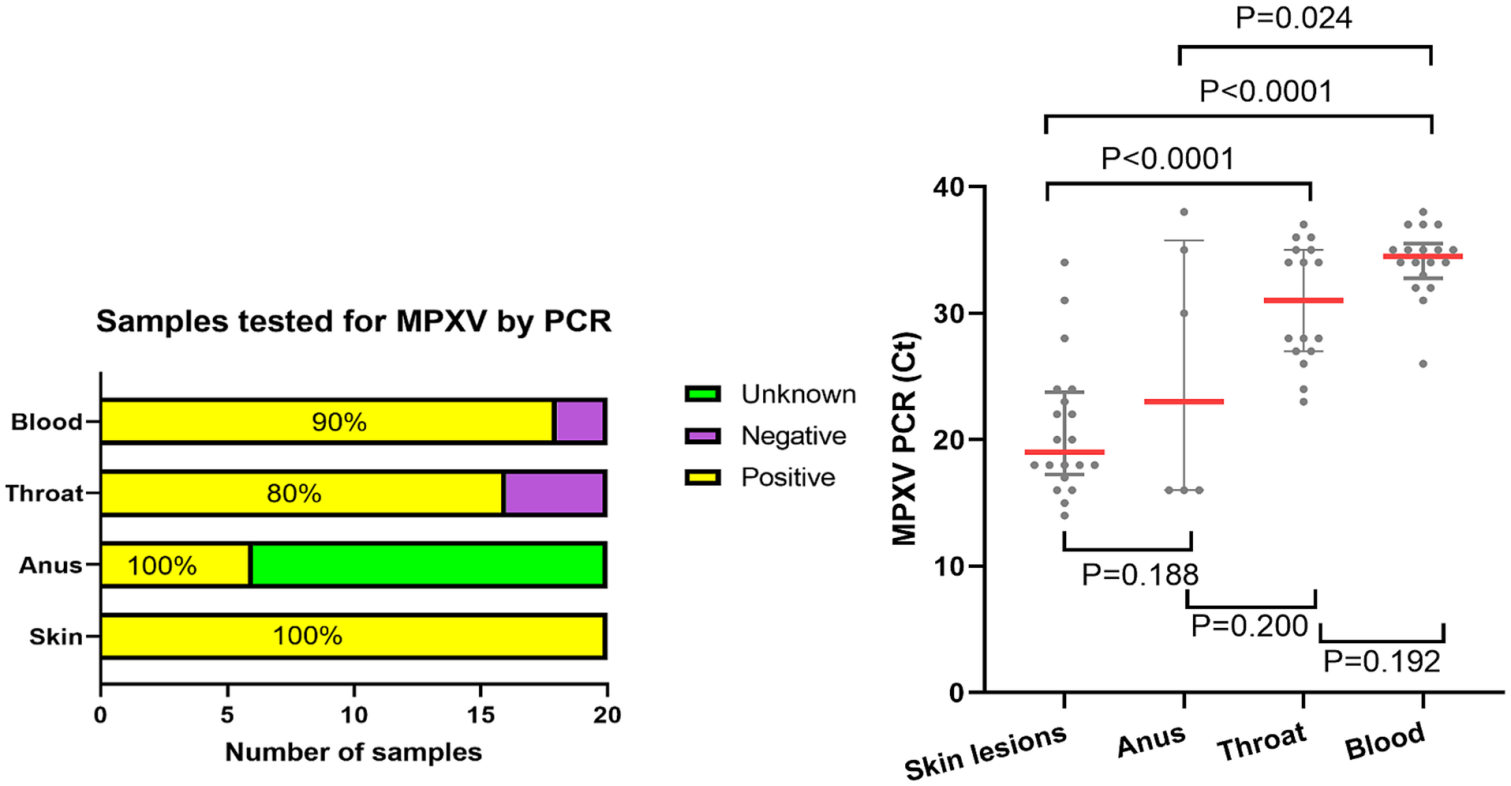
Monkeypox viral loads, given as PCR Ct values, across the different anatomical areas. Monkeypox virus (MPXV) detection showed a higher positive rate in skin [20 (100%) of 20] and anal [6 (100%) of 6] samples compared to those from blood [18 (90%) of 20] or throat [16 (80%) of 20] **(A)**. Cycle threshold (Ct) value was significantly lower in skin lesions (Ct value = 19) than in the throat (Ct value = 31) or blood (Ct value = 35) lesions (*p* < 0.001). Ct value from anal samples (Ct value = 23) was lower than those from blood (Ct value = 35) (*p* < 0.05) **(B)**. The Ct value indicates the number of PCR cycles required to detect MPXV, with a higher Ct indicating lower levels of viral DNA, and a cutoff of 40 cycles representing undetectable DNA.

PCR tests were performed for all 20 skin lesions and throat samples: 20 positive (100%) skin lesions, with a median Ct-value of 19 (IQR: 17–24), and 16 positive (80%) and four negative (20%) throat samples, with a median Ct-value of 31 (IQR: 27–35). Blood PCR tests were also conducted for all 20 patients; among them 18 were positive (90%) and two negative (10%), with a median Ct-value of 35 (IQR: 33–36). PCR testing of anal samples was performed for six patients: six samples were positive (100%), with a median Ct-value of 23 (IQR: 16–35) (Figure 5A). The Ct values from the skin lesions (Ct value = 19) were significantly lower compared to those from the throat (Ct value = 31) and blood (Ct value = 35) (*p* < 0.001) samples. The Ct value of the anal samples (Ct = 23) was lower than that of the blood samples (Ct = 35) (*p* < 0.05) (Figure 5B).

Patients presented with various comorbidities, including depression and insomnia, which may have been due to either MPX infection or the stress of being in an isolation facility. They also experienced pain due to skin lesions or lymphadenopathy; and skin and soft tissue infections. The treatment regimen primarily included pain relief medications and oral antibiotics for skin and soft tissue infections. No significant biochemical or hematological abnormalities were observed. None of the patients were immunocompromised and no severe cases were observed. All patients ultimately achieved a full recovery. Further, none of the healthcare workers who cared for these patients became ill.

## 4 Discussion

In this study, we report 20 cases of human MPX infection diagnosed in China between June 10, 2023, and July 15, 2023, amidst the ongoing MPXV outbreak. Our findings include data on MPX co-infections with HIV and syphilis, as well as MPXV loads in various anatomical sites, including the skin, throat, anus, and blood, at the time of diagnosis. This highlights the prevalence of STIs among MSM with MPXV infections and the correlation between sexual practices and the acquisition of MPXV.

The most affected patients in our cohort were young adult men in their 20s. As reported in European countries, MPX transmission mainly occurs in MSM populations (5, 6) and patients sexually infected with MPXV present with localized anogenital lesions, with some spread to distant lesions (involving the trunk, face, and limbs); however, MPXV rarely disseminates throughout the body (7). In this study, all affected patients were MSM. Notably, 85% of patients had anogenital lesions, 60% had trunk and limb lesions, 35% had face and mouth lesions, and 5% had head lesions. The location of the lesions (mainly in the perianal or genital regions) may be associated with the manners of sexual activity or intimate contact (8).

In West and Central Africa, MPX mainly affects older children, adolescents, and young adults (9). In this study, however, MPXV mainly affected young men. Notably, 19 (95%) patients reported having unprotected sexual encounters with men in the month preceding their diagnosis. This finding aligns with a 2022 study conducted across 16 countries, which found that 95% of MPXV infections were transmitted sexually, with a 98% prevalence among MSM. This suggests that transmission of MPXV during sexual intercourse and high-risk sexual behaviors are potential risk factors (5). Notably, our study included one confirmed case where the only reported contact was a kiss, with no sexual encounter in the preceding month. This suggests that close contact with body fluids might also transmit the infection. A case reported in 2018, in the United Kingdom, showed MPX transmission from a patient to a healthcare employee, likely linked to contact with contaminated bedding (10). In addition, transmission can occur via close contact, including direct contact with an MPX rash or scabs, fomites, or exposure to the respiratory secretions of an infected individual (11, 12).

In this study, 50% of the confirmed patients had syphilis and 65% had HIV. Notably, eight (40%) patients had HIV and syphilis co-infection. Concomitant STIs have been documented in 16–29% of cases in published cohorts, with gonorrhea, chlamydia, and syphilis being the most common infections (13). Two recent studies from Madrid and London, respectively, reported similarly high rates of STIs among MSM with MPXV infections (14, 15), suggesting that health agencies should prioritize the importance of testing for other STIs and obtaining a detailed sexual history.

During the multi-county 2022 MPX outbreak, a highly significant association with HIV was noted, with the United States Centers for Disease Prevention and Control reporting a HIV co-infection rate of 46% in MPX cases and an HIV prevalence of 28–47% in other countries’ reports among patients with MPXV infection (4, 13, 15–18). In our study, the prevalence of HIV infection was 65%. Similar to other published data in men, the median CD4+ T lymphocyte cell count was high (667 cells/mm^3^, IQR, 404–902) among the 13 patients with HIV, and 12 (92%) were on antiretroviral treatment. The patients were not immunocompromised and did not experience any complications. The clinical spectrum showed no apparent differences between individuals with and without an HIV infection. The observed clinical characteristics did not differ from those previously reported (13). However, severe MPX cases and even deaths have been reported, particularly in immunocompromised patients, including those with advanced untreated HIV infection (19–21). For instance, an MPX-related death in the US involved a 33-year-old man with syphilis and HIV co-infection, who had a CD4+ T cell count of <35 cells/mm^3^ (21). HIV patients with high CD4+ T-cell counts (>350 cells /mm^3^) may benefit from a similar poxvirus-specific T-cell response (20); however, for cases with low CD4+ T-cell counts, close monitoring of the potential risk of developing more severe illness is necessary.

Our findings indicated that MPXV loads were significantly higher in skin (Ct = 19) and anal lesions (Ct = 23) compared to those in the throat (Ct = 31) or blood samples (Ct = 35). This aligns with the results from Mitjà’s study, which found that samples from skin lesions contained substantially more viral DNA than those from the throat (7). Indeed, several studies have demonstrated that viral loads in body fluids are generally lower than in skin lesions (10, 22). High MPXV loads in skin lesions, especially from the genital and anal sites, suggests that MPX transmission most likely occurs through direct body contact rather than through the respiratory route or contact with body fluids, which helps refine the preventive measures delivered to individuals exposed to MPXV (23, 24). Furthermore, our study indicates that blood samples may not be the most suitable for PCR assays in diagnosing MPX, since viremia is limited by the timing of sample collection after the onset of symptoms.

The main limitations of our study include the small sample size and its monocentric nature, which could introduce patient recruitment bias. This limitation raises the possibility that individuals with milder symptoms or without symptoms may have been missed. Consequently, further studies are required to establish a more definitive association between MPX and STIs to support our conclusions. Yet, despite the small sample size, this study contributes valuable data from China on MPXV infections during the current outbreak. Moreover, we did not test our patients for other sex transmitting diseases pathogens, except for HIV and syphilis. Papers from other countries reported that herpes simplex virus type 2 and varicella-zoster virus may also be closely associated with MPX (9, 25, 26). Data on whether patients with MPX were co-infected with other sex transmitting diseases pathogens were not available in our study. Thus, future studies should focus on gathering and studying detailed data on viral load kinetics and the duration and infectiousness of viral shedding in bodily fluids.

## 5 Conclusion

This study showed a high percentage of MPX co-infection with HIV and syphilis, highlighting the value of testing for STIs, especially HIV and syphilis, at the time of MPX suspicion or diagnosis. Therefore, a rapid and comprehensive educational movement for at-risk populations in response to MPX is urgently required to prevent its transmission.

## Data availability statement

The datasets presented in this study can be found in online repositories. The names of the repository/repositories and accession number(s) can be found in the article/supplementary material.

## Ethics statement

The studies involving humans were approved by the Ethics Committee of Beijing Youan Hospital (China) (approval number: LL-2023-035-K). The studies were conducted in accordance with the local legislation and institutional requirements. The participants provided their written informed consent to participate in this study. Written informed consent was obtained from the individual(s) for the publication of any potentially identifiable images or data included in this article.

## Author contributions

LJ: Conceptualization, Data curation, Formal analysis, Investigation, Methodology, Writing – original draft, Writing – review & editing. BY: Investigation, Validation, Writing – review & editing. YF: Investigation, Validation, Writing – review & editing. XY: Investigation, Validation, Writing – review & editing. HJ: Investigation, Validation, Writing – review & editing. MZ: Investigation, Validation, Writing – review & editing. SL: Writing – review & editing, Investigation. YZ: Writing – review & editing, Supervision. WW: Supervision, Writing – review & editing. CG: Supervision, Writing – review & editing. TZ: Writing – review & editing, Funding acquisition. XH: Conceptualization, Funding acquisition, Supervision, Writing – review & editing. TJ: Conceptualization, Formal analysis, Supervision, Writing – review & editing.
